# Ruthenium(II) Complex-Based Tetradentate Schiff Bases: Synthesis, Spectroscopic, Antioxidant, and Antibacterial Investigations

**DOI:** 10.3390/ijms25147879

**Published:** 2024-07-18

**Authors:** Bouchra Es-Sounni, Kaoutar Harboul, Ayoub Mouhib, Ashwag S. Alanazi, Mohamed Hefnawy, Mohamed Bakhouch, Taoufiq Benali, Khalil Hammani, Noureddine Mazoir, Mohamed El Yazidi, Ahmed Benharref, Mohammed Fahim

**Affiliations:** 1 Laboratory of Innovative Materials and Biotechnology of Naturel Resources, Faculty of Sciences, Moulay Ismail University, Meknes 50000, Morocco; bouchrasounni@gmail.com (B.E.-S.);; 2 Laboratory of Natural Resources and Environment, Polydisciplinary Faculty of Taza, Sidi Mohamed Ben Abdellah University of Fez, Taza 30050, Moroccobenali.taoufiq@gmail.com (T.B.); khalil.hammani@usmba.ac.ma (K.H.); 3 Bioorganic Chemistry Team, Department of Chemistry, Faculty of Sciences, Chouaïb Doukkali University, El Jadida 24000, Morocco; 4 Department of Pharmaceutical Sciences, College of Pharmacy, Princess Nourah Bint Abdulrahman University, Riyadh 1167, Saudi Arabia; asalanzi@pnu.edu.sa; 5 Department of Pharmaceutical Chemistry, College of Pharmacy, King Saud University, Riyadh 11451, Saudi Arabia; mhefnawy@ksu.edu.sa; 6 Environment and Health Team, Polydisciplinary Faculty of Safi, Cadi Ayyad University, Safi 46000, Morocco; 7 Engineering Laboratory of Organometallic and Molecular Materials and Environment, Faculty of Sciences Dhar El Mahraz, University Sidi Mohamed Ben Abdellah, Fez 30000, Morocco; elyazidimohamed@hotmail.com; 8 Laboratory of Natural Substances Chemistry, Faculty of Sciences Semlalia, Cadi Ayyad University, Marrakech 40000, Morocco; a.benharref@gmail.com

**Keywords:** salen Schiff bases, Ru(II) complexes, spectroscopy, antioxidant, antibacterial

## Abstract

In this work, we describe the synthesis of novel Ruthenium (II) complex-based salen Schiff bases. The obtained Ruthenium (II) complexes are characterized using usual spectroscopic and spectrometric techniques, viz., IR, UV-Vis, NMR (^1^H and ^13^C), powder X-ray diffraction, and HRMS. Further techniques, such as DTA-TGA and elemental analysis, are used to well establish the structure of the obtained complexes. Octahedral geometries are tentatively proposed for the new Ru(II) complexes. The measured molar conductance for the Ruthenium (II) complexes shows their electrolytic nature (4.24–4.44 S/m). The new Ru(II) complexes are evaluated for their antioxidant and antibacterial activities. The DPPH radical scavenging, FRAP, and total antioxidant capacity (TAC) assays show that the obtained complexes are more potent than the used positive control. They also exhibit promising antibacterial responses against pathogen bacteria: [RuH_2_L^3^Cl_2_] exhibits an important inhibition against *Bacillus subtilis* DSM 6633, with an inhibition zone of 21 ± 1.41 mm with an MIC value of 0.39 mg/mL, and *Proteus mirabilis* INH, with 16.50 ± 0.70 mm and an MIC value of 0.78 mg/mL, while [RuH_2_L^2^Cl_2_] exerts interesting antibacterial effects versus *Bacillus subtilis* DSM 6633 (21 ± 1.41 mm) and *Proteus mirabilis* INH (25.5 ± 0.70 mm) with equal MIC values of 0.97 mg/mL.

## 1. Introduction

The chemistry of ruthenium has attracted considerable interest due to the large array of properties manifested by the complexes of this metal [1,2,3]. Indeed, they have been widely used as catalysts [4,5] and as sensitizers in dye-sensitized solar cells [2,3]. Ruthenium complexes were also found to have specific biological activities like anticancer [6,7,8], antimicrobial [9], and antioxidant [10,11] activities. They have also shown an interesting catalytic activity for several chemical reactions [12,13]. The properties observed for the ruthenium complexes are dependent on the environment around the central metal ion, hence, the complexation of ruthenium with different ligands could lead to interesting complexes endowed with a large spectrum of applications [14].

**Figure 1:**
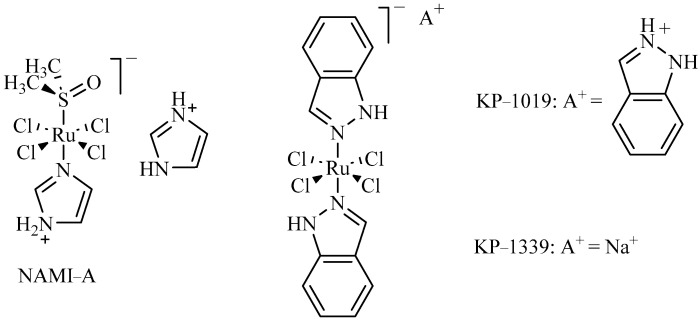
NAMI−A and KP−1019/KP−1339 used as clinical trials.

In 1980, Yasbin et al. [15] studied the anticancer activity of the first ruthenium complex (cis-Ru(NH_3_)_4_(Cl)_2_). Since then, ruthenium complexes have been screened against several cancer cell lines and have shown promising therapeutic effects [1]. Indeed, ruthenium complexes, namely, NAMI−A and cis-[Ru(Cl)_4_(indazole)_2_] or KP−1019/KP−1339 (Figure 1), have successfully entered phase-II clinical trials [16,17]. In particular, ruthenium complexes are generally recognized as the least toxic and most efficient against the resistance induced by platinum drugs in cancerous cells [18]. Hence, ruthenium complexes could be considered as promising alternative drug candidates for cisplatin and its derivatives.

Based on the above-mentioned statements, we have decided to report the synthesis of three Ruthenium (II) complex-based salen Schiff bases. The obtained Ru(II) complexes have been screened for their antioxidant activity using DPPH and FRAP assays. The antibacterial activity has been also evaluated for the studied complexes against pathogen bacteria including Gram-positive bacteria (*Bacillus subtilis* DSM 6633 and *Staphylococcus aureus* CECT 976) and Gram-negative bacteria (*Proteus mirabilis* INH, *Escherichia coli* K12, and *Pseudomonas aeruginosa* CECT 118).

## 2. Results and Discussion

**Scheme 1:**
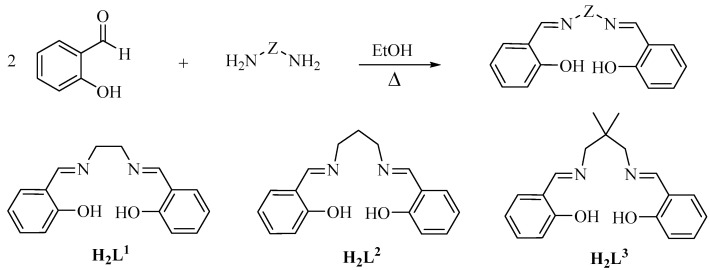
Synthetic pathway of salen Schiff base ligands.

The synthesis of the base Schiff ligands was performed using the slightly modified reported procedure [19]. The appropriate alkane-diamine was condensed on two moles of salicylaldehyde in refluxed ethanol (Scheme 1).

**Scheme 2:**
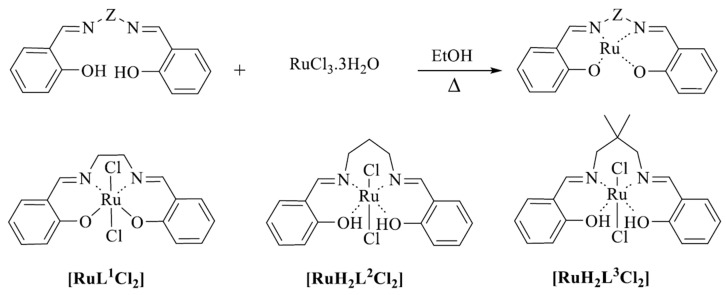
Synthesis of Ru(II) complexes.

The obtained ligands were then reacted with RuCl_3_.3H_2_O salts in alcohol at reflux using the described method in the literature [20]. The Ru(II) complexes were obtained in excellent yield (Scheme 2).

### 2.1. Spectroscopic Studies

The IR spectra of the salen ligands and their Ru(II) complexes are given in the supplementary content file (Figures S1–S6). The FT-IR spectra of the free ligands show the existence of the characteristic bands between 1628 and 1632 cm^−1^ attributed to the stretching vibrations (C=N) and (C=C). These bands are shifted to lower frequencies (1538–1628 cm^−1^) in the spectra of the complexes, in addition, the bands related to the vibration of the O-H bond observed at the range 3423–3450 cm^−1^ for the free ligands have shifted to lower frequencies in [RuH_2_L^2^Cl_2_] and [RuH_2_L^3^Cl_2_] spectra, indicating that the iminic nitrogen and phenolic oxygen of the Schiff base are coordinated to the Ru(II) ion. This observation leads to the conclusion that the formation of the complexes takes place without deprotonation of the hydroxyl group of the salen ligands, whereas the IR spectrum of the [RuL^1^Cl_2_] complex shows a band at 3434 cm^−1^ attributed to the stretching of O-H bond, showing that this complex is formed without deprotonation of the hydroxyl group.

The ^1^H-NMR spectra of the salen ligands H_2_L^1^, H_2_L^2^, and H_2_L^3^ (Figures S7, S9 and S11) recorded in deuterated chloroform show a singlet signal at around 8.35 ppm integrating to the protons of the azomethine groups (CH=N) and a signal at around 13.60 ppm integrating the protons corresponding to the hydroxyl groups (OH). The ^1^H-NMR spectra also show the presence of a singlet around 3.51 ppm attributable to protons from methylene groups adjacent to the azomethine group (-CH_2_-HC=N) and another singlet signal at 1.10 ppm corresponding to methyl protons. The spectrum revealed the absence of the signal between 4 ppm and 6 ppm relating to the protons of the diamine NH_2_ function. In addition, their ^13^C-NMR spectra (Figures S8, S10 and S12) show show a signal at range of 56.83–68.13 ppm attributed to the carbon of methylene groups adjacent to the nitrogen atom (-CH_2_-N=C). They show also a signal around 161 ppm corresponding to the quaternary carbon linked to the hydroxy goup (C-OH). The carbon of azomethine groups resonate around 166 ppm. Thus, the NMR spectral data are in good agreement with the proposed structure of the salen ligands for each case studied.

However, the ^1^H-NMR spectra of the Ru complexes (Figures S13 and S15) with those of the base ligands can show that the resonance signal attributed to the hydroxyl proton in the free ligands has disappeared in the ^1^H-NMR spectra of the [RuL^1^Cl_2_] complex, which explains the deprotonation of H_2_L^1^ in the complexation with Ru(II). The ^1^H-NMR spectra of [RuH_2_L^3^Cl_2_] attach a resonance of the hydroxyl proton, endorsing that complexation, in this case, is without deprotonation. The ^13^C-NMR spectra of [RuL^1^Cl_2_] and [RuH_2_L^3^Cl_2_] complexes (Figures S14 and S16) display all characteristic signals belonging to the appropriate carbons of the proposed structures. To further corroborate the proposed structure for the synthesized complexes, we measured their HRMS spectra using ESI^+^ ionization. The HRMS spectra (Figures S17–S19) of the Ru(II) complexes exhibit *m*/*z* peaks that match the [M+H]^+^ of the Ru complexes and support their suggested structures.

### 2.2. UV-Vis Spectra

**Figure 2:**
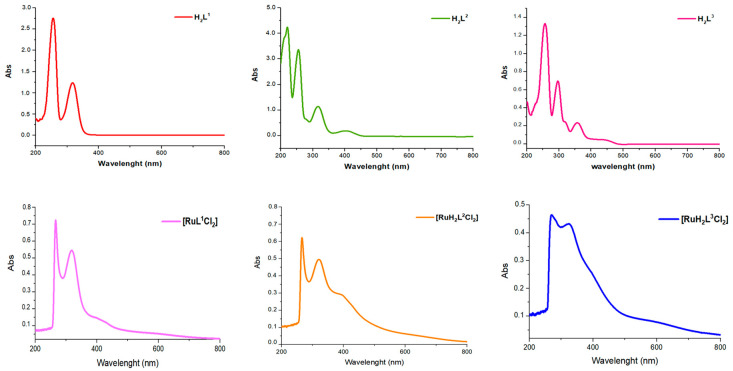
UV-Vis spectra of salen ligands and their Ru(II) complexes.

The UV-Vis spectra of ligands and their Ru complexes are presented in Figure 2. Four clear bands are exhibited for the ligands, the two first intense absorption bands that appeared in the range 219–296 nm are due to the (π–π*) electronic transition, while the third absorption band between 315 and 355 nm is due to (n→π*) transitions, and the low intense band located at a range of 398–447 nm is assigned to the ligand–metal charge transfer transitions. The spectra of Ru(II) complexes show that the bands related to (n→π*) electronic transition of the free Schiff base ligands exhibit a redshift. This could be explained by the coordination of the azomethine moiety following the donation of its non-binding electron pair to the Ru(II) ion [16]. In addition, the π→π* transitions of the free ligands also undergo a redshift in the Ru complexes’ spectra. In addition to the bands shifted to longer wavelengths, the complexes’ spectra show a new band between 600 and 650 nm attributed to d-d transitions. All these changes attest to the coordination of the Ru(II) ion with the salen Schiff base ligands.

### 2.3. X-ray Powder

**Figure 3:**
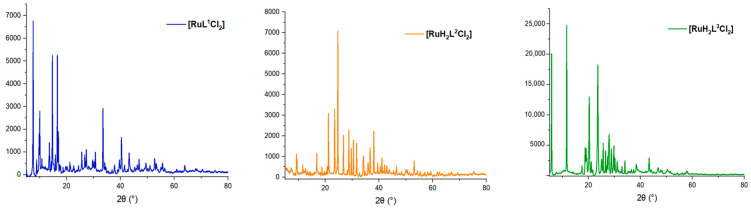
X-ray diffraction patterns of Ru(II) complexes.

The Ru(II) complexes were analyzed by X-ray powder diffraction. The diffractograms of the compounds are shown in Figure 3, which show the presence of sharp peaks indicating that the synthesized Ru(II) complexes have a crystalline nature. The peaks appeared to be very intense, indicating the high crystallinity of all the compounds analyzed.

The obtained diffraction data, shown in Table 1, reveal that all the Ru complexes have the same monoclinic crystal system.

### 2.4. Thermal Analysis

**Figure 4:**
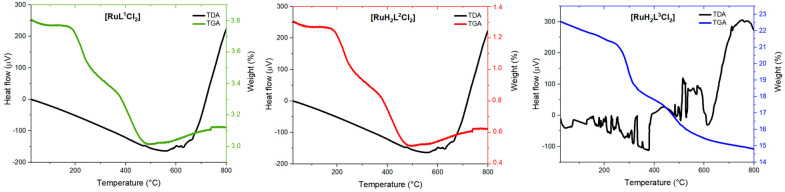
DTA/TGA thermograms of Ru(II) complexes.

The thermal stability of the synthesized ligands and their corresponding Ru(II) complexes was studied using TGA and DTA methods over a temperature range from room temperature up to 1000 °C. The thermograms of the compounds (Figure 4) show that all the Ru(II) complexes are stable up to 400 °C. However, the thermal decomposition of the salen ligands starts at around 300 °C. The absence of a relative loss of hydrated and complexed water molecules shows that the complexes are not hydrated. The decomposition of the residual complexes occurred at higher temperatures between 400 and 625 °C, demonstrating that the obtained Ru(II) complexes are highly stable.

### 2.5. Antioxidant Activities Essays

**Figure 5:**
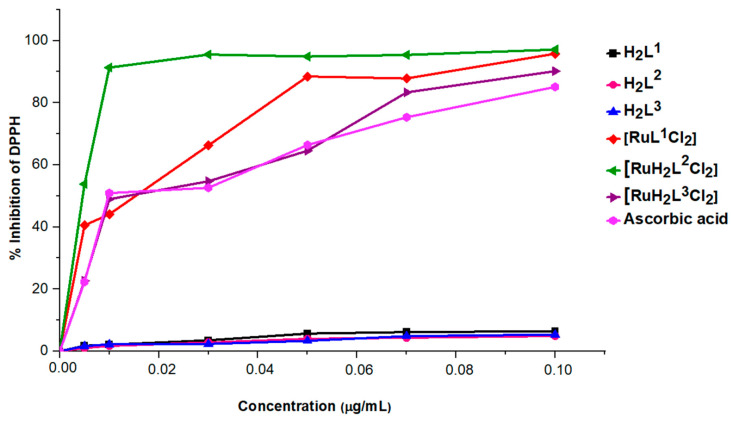
DPPH radical scavenging activity for salen ligands and their Ru(II) complexes.

The antioxidant power of the salen ligands and their Ru complexes was evaluated using the DPPH free radical scavenging (Figure 5). The obtained results show that all the tested compounds are active and can scavenge free radicals. The study proved that Ru(II) complexes are more effective in preventing the formation of radicals than free ligands, which explains why the complexation of salen by Ru favored antioxidant power. These results could be due to the variety of oxidation degrees of the Ru ion, which allows it to accept or give up an electron easily and ultimately neutralizes the DPPH radical. In addition, the choice of salen-bridging ligands protects Ru ions from undesirable interactions and enhances effective interaction with free radicals. Furthermore, the Ru(II) complexes show a greater capacity to neutralize free radicals than the ascorbic acid used as a standard, making it a potentially more effective candidate for combating oxidative damage.

The antioxidant capacity of the various compounds was determined from the IC_50_ values. It is the concentration required to reduce 50% of the DPPH radical. The lower the IC_50_ value, the greater the antioxidant activity of a compound. For each compound, we determined the concentration required to reduce 50% of the DPPH free radical, or IC_50_, from the linear regression equations of the graphs. The values are shown in Table 2.

According to the obtained results in Table 2, the values of IC_50_ obtained for the complexes [RuL^1^Cl_2_] (0.09 ± 0.03 μg/mL) and [RuH_2_L^3^Cl_2_] (0.04 ± 0.01 μg/mL) are the lowest of all the tested compounds, even that of ascorbic acid (0.14 ± 0.01 μg/mL) used as a standard, therefore, they reflect their very important antioxidant activity. The third complex [RuH_2_L^2^Cl_2_] has a low IC_50_ value (0.15 ± 0.03 μg/mL), close to that of ascorbic acid, which also shows its strong reducing power.

**Figure 6:**
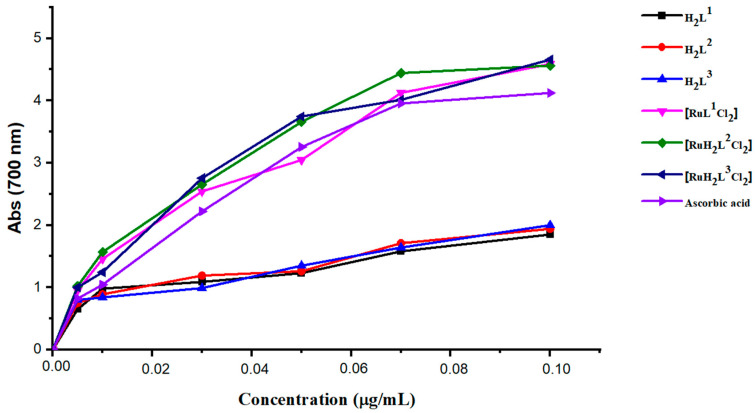
Antioxidant activity-FRAP assay for salen ligands and their Ru(II) complexes.

According to the FRAP assay, the results in Figure 6 show that the Ru complexes exhibit a greater reduction capacity of the ferric ion to the ferrous ion than the free ligands. The outcomes show also that the Ru complexes are more active than ascorbic acid used as the reference antioxidant agent.

**Figure 7:**
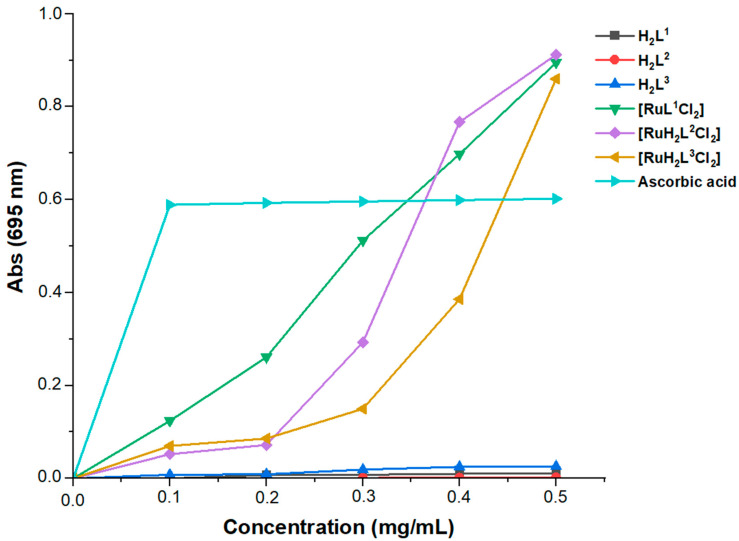
Antioxidant activity of Ru(II) complexes and their based salen ligands.

To assess the antioxidant activity of salen ligands and their Ru complexes, the ammonium phosphomolybdate method was used. In this method, the free radicals generated react with the antioxidants (tested compounds), reducing the concentration of free radicals and causing a change of color in the reactive solution, generally towards a blue hue. The more intense the blue color, the higher the antioxidant activity of the sample. According to the results obtained (Figure 7), the base ligands showed a very low reduction capacity, but their Ru complexes showed strong efficiency in reducing Mo(VI) to Mo(V). In addition, the Ru complexes showed moderate activity compared with ascorbic acid (standard) for concentrations below 0.3 mg/mol; but above this concentration, they exerted high reduction better than that of ascorbic acid.

The observed outcomes show that the Ru complexes exhibit excellent antioxidant activities compared to the free ligand, which are in good agreement with the previously reported work on analogous Ru complexes [21]. In addition, it is important to note that our results indicate that the synthesized Ru complexes display strong reducing capacities, validated by the IC_50_ values obtained for the DPPH assay. Similarly, the results observed for the FRAP assay demonstrate very high absorbance values, higher than those previously reported [22].

### 2.6. Antibacterial Activity

The results of the in vitro tests of the antibacterial effect of all products, using the filter paper disc diffusion and the microdilution methods against selected microorganisms, are illustrated in Table 3. Among the Gram-negative and Gram-positive tested bacteria, *P. mirabilis* and *B. subtilis* are the most sensitive to the screened products. For *P. mirabilis*, the highest antibacterial activity was exhibited by [RuH_2_L^2^Cl_2_] with a diameter of inhibition zones of 25.5 ± 0.70 mm, followed by [RuH_2_L^3^Cl_2_] (16.50 ± 0.70 mm). Concerning *B. subtilis*, [RuH_2_L^2^Cl_2_] inhibits its growth with an important inhibition zone of 21 ± 1.41 mm, followed by [RuH_2_L^3^Cl_2_] (17 ± 1.41 mm), while no antibacterial activity was obtained by [RuL^1^Cl_2_]. The MBC/MIC values inform that [RuH_2_L^2^Cl_2_] and [RuH_2_L^3^Cl_2_] exert a bactericidal effect against *P. mirabilis* and *B. subtilis*. On the other hand, our results demonstrated that *E. coli* K12, *P. aeruginosa* CECT 118, and *S. aureus* CECT 976 are resistant to the tested products. The obtained results in this study show that the Ru complexes exhibit promising antibacterial activity which are in agreement with the literature [23,24], which has shown that Ru complexes exhibit strong antibacterial activity against many pathogenic strains.

## 3. Materials and Methods

### 3.1. Experimental

RuCl_3_.3H_2_O was purchased from Sigma-Aldrich and used as supplied. Solvents were of analytical grade and used without any further purification. Infrared spectra were recorded at room temperature using a Thermo Scientific ^TM^ Nicolet iS10 FT-IR spectrometer, ranging from 400 to 4000 cm^−1^. ^1^H and ^13^C NMR spectra were recorded at room temperature using the BRUKER AVANCE II 300 MHz and 500 MHz JEOL (JNM-ECZ500R/S1 FT NMR) Systems. The deuterated solvents used to record NMR spectra are mentioned with the spectral data for each compound. X-ray analysis diffraction was performed on a Bruker advance D8 eco diffractometer (50 KV–20 mA) equipped with a CuKα radiation source (λ = 1.5418 Å). The HRMS spectra were recorded using Waters^®^ AcquityTM Ultra Performance LC coupled to an AcquityTM triple quadrupole (Waters^®^, Wexford, Ireland) and equipped with an electrospray ionization (ESI) source operating in both positive and negative ion modes. The electronic spectra were recorded in ethanol using a UV-6300PC/VWR spectrophotometer. The melting temperature was measured on a Kofler Bench. Schiff base ligands H_2_L^1^, H_2_L^2^, and H_2_L^3^ were prepared using a slightly modified previously described procedure [25,26,27].

### 3.2. General Procedure for the Preparation of Ligands

In a 100 mL round bottom flask equipped with a condenser, appropriate diamine (1 mmol), salicylaldehyde (2 mmol), and absolute ethanol (10 mL) were introduced. The mixture was refluxed under magnetic stirring and the evolution of the reaction was monitored by thin-layer chromatography (TLC). When TLC indicates the consumption of reagents, the solvent was evaporated by a rotary evaporator, and the obtained crude product was crystallized in ethanol. The yellow precipitate was isolated by filtration, washed with cold ethanol, and dried under a vacuum.

*N,N’-bis(salicylidene)-1,2-diaminoethane (H_2_L^1^)*

Yellow crystals; 94% yield; m.p. = 80 °C.

ATR-IR: ν(O-H): 3377 cm^−1^; ν(C=N): 1632 cm^−1^; ν(C=C): 1418, 1454, 1496, 1532 cm^−1^; ν(C–O): 1281 cm^−1^. ^1^H NMR (CDCl_3_, 300 MHz, δ in ppm): 3.93 (s, 4H, (CH_2_)_2_), 6.88 (dt, 2H, ^3^J = 7.47 Hz, ^4^J = 0.78 Hz), 6.97 (d, 2H, J_o_ = 7.99 Hz), 7.24 (dd, 2H, J_o_ = 7.66 Hz, J_m_ = 1.58 Hz), 7.32 (dt, 2H, Ar-H, J_o_ = 7.54 Hz, J_m_ = 1.58 Hz), 8.36 (t, 2H, -CH=N), 13.25 (s, 2H, Ar–OH). ^13^C NMR (CDCl_3_, 75 MHz, δ in ppm): 59.73 ((CH_2_)_2_), 116.96, 118.6 (-C-CH=N), 118.7, 131.5, 132.5, 161.0 (-C-OH), 166.5 (-CH=N). UV-Vis (DMSO, λ_max_ in nm (ɛ_max_ in L·mol^−1^·cm^−1^)): 271 (15917), 316 (13244), 398 (2731).

*N,N’-bis(salicylidene)-1,3-diaminopropane (H_2_L^2^)*

Yellow solid; 90% yield; m.p. = 80 °C.

ATR-IR: ν(O-H): 3253 cm^−1^; ν(C=N): 1628 cm^−1^; ν(C=C): 1417, 1437, 1456, 1496 cm^−1^; ν(C–O): 1280 cm^−1^. ^1^H NMR (CDCl_3_, 300 MHz, δ in ppm): 2.13 (q, 2H, CH**_2_**(CH_2_)_2_, *J* = 6.6 Hz ), 3.73 (t, 4H,CH_2_(CH**_2_**)**_2_**, *J* = 6.6 Hz), 6.91(dt, 2H, Ar-H, *J_1_* = 1.2 Hz, *J_2_* = 7.8 Hz), 7.00 (d, 2H, Ar-H, *J* = 8.4 Hz), 7.27 (dd, 2H, Ar-H, *J*_1_ = 1.8 Hz, *J*_2_ = 7.8 Hz), 7.34 (dt, 2H, Ar-H, *J*_1_ = 1.5 Hz, *J*_2_ = 7.5 Hz), 8. 39 (s, 2H, -CH=N), 13.47 (s, 2H, Ar–OH). ^13^C NMR (CDCl_3_, 75 MHz, δ in ppm): 31.7 (CH_2_(CH_2_)_2_), 56.8 2(CH_2_-N=CH), 117.0, 118.6, 118.7 (-C-CH=N), 131.3, 132.3, 161.1 (-C-OH), 166.4 (-CH=N). UV-Vis (DMSO, λ_max_ in nm, (ɛ_max_ in L·mol^−1^·cm^−1^)): 219 (42501), 256 (33711), 315 (11579), 403 (2100).

*N,N’-bis(salicylidene)-2,2-dimethyl-1,3-diaminopropane (H_2_L^3^)*

Yellow solid; 95% yield; m.p. = 57 °C.

ATR-IR: ν(O-H): 3379 cm^−1^; ν(C=N): 1632 cm^−1^; ν(C=C): 1418, 1453, 1496, 1532 cm^−1^; ν(C–O): 1281 cm^−1^. ^1^H NMR (CDCl_3_, 300 MHz, δ in ppm): 1.10 (s, 6H, (CH**_3_**)**_2_**); 3.51 (d, 4H, (CH**_2_**)**_2_**, ^4^J = 1.07 Hz); 6.91 (dt, 2H, Ar-H, J_m_ = 1.06 Hz, J_o_ = 7.50 Hz); 7.00 (d, 2H, Ar-H, J = 8.08 Hz); 7.28 (dd, 2H, Ar-H, J_m_ = 1.63 Hz, J_o_ = 7.66 Hz); 7.34 (dt, 2H, Ar-H, J_m_ = 1.70 Hz, J_o_ = 7.35 Hz); 8.35 (s, 2H, CH=N), 13.60 (s, 2H, Ar–OH). ^13^C NMR (CDCl_3_, 75 MHz, δ in ppm): 24.49 ((CH_3_)_2_), 36.26 (C(CH_3_)_2_), 68.13 ((CH_2_)_2_), 117.06, 118.59, 118.74 (C-CH=N), 131.38, 132.65, 161.22 (C-OH), 166.05 (CH=N). UV-Vis (DMSO, λ_max_ in nm, (ε_max_ in L·mol^−1^·cm^−1^): 256 (13425), 296 (7103); 355 (2390); 447 (575).

*General procedure for the synthesis of ruthenium complexes*

In 100 mL flasks equipped with a condenser and dropping funnel, the appropriate ligand (1 mmol) was dissolved in ethanol (20 mL). Then, a solution of RuCl_3_.3H_2_O (1 mmol) in 2 mL of ethanol was added dropwise through a dropping funnel. The mixture was refluxed under magnetic stirring for 6 h. After, the mixture was cooled to room temperature, and the formed precipitate was isolated by filtration and washed with diethyl ether and cold ethanol. The solid was recrystallized in ethanol to obtain a pure complex.

*[RuL^1^Cl_2_]: Dark green solid; 78% Yield.*

ATR-IR: ν (CH=N): 1627 cm^−1^; ν(C=C): 1416, 1434, 1459, 1496 cm^−1^; ν(C–O): 1277 cm^−1^; ν(O-Ru): 663 cm^−1^; ν(N-Ru): 642 cm^−1^. ^1^H NMR (DMSO-d_6_, 500 MHz, δ in ppm): 2.92 (t, 2H, CH_2_), 3.47 (t, 2H, CH_2_), 7.08 (dd, 1H, Ar-H, J_o_ = 5.05 Hz, J_m_ = 4.47), 7.31 (d, 2H, Ar-H, J_o_ = 7.94), 7.40 (dd, 1H, Ar-H, J_o_ = 3.64, Hz, J_m_ = 1.18 Hz), 7.62 (t, 3H, Ar-H, J_o_ = 5.02 Hz), 7.67 (s, 1H, Ar-H), 8.30 (s, 1H, -CH=N). ^13^C NMR (DMSO, 75 MHz, δ in ppm): 43.69 (CH_2_), 59.87 (CH_2_), 127.05, 128.25, 130.14, 132.11 (-C-CH=N), 156.74 (-CH=N). UV–Vis (DMSO, λ_max_ in nm, (ε_max_ in mol·L^−1^·cm^−1^): 265 (6278), 319 (4950), 401 (2901), 644 (609). HRMS [M+H]^+^: calcd: 437.94758, obtained: 438.02587. Anal. Calcd for C_16_H_14_N_2_O_2_Cl_2_Ru: C, 43.85; H, 3.22; N, 6.39; O, 7.30. Found: C, 43.11; H, 3.10; N, 6.27; O, 7.02.

*[RuH_2_L^2^Cl_2_]: Dark green solid; 69% Yield.*

ATR-IR: ν(O-H): 3390 cm^−1^; ν(CH=N): 1638 cm^−1^; ν(C=C): 1404, 1425, 1459, 1516 cm^−1^; ν(C–O): 1277 cm^−1^; ν(O-Ru): 629 cm^−1^; ν(N-Ru): 603 cm^−1^. UV–Vis (DMSO, λ_max_ in nm, (ε_max_ in mol·L^−1^·cm^−1^): 269 (4678), 325 (4330); 406 (2548); 600 (874). HRMS [M+H]^+^: calcd: 453.97888, obtained: 454.07856. Anal. Calcd for C_16_H_14_N_2_O_2_Cl_2_Ru: C, 44.94; H, 3.99; N, 6.17; O, 7.04. Found: C, 44.01; H, 3.51; N, 6.12; O, 7.19.

*[RuH_2_L^3^Cl_2_]: Dark green solid; 84% Yield.*

ATR-IR: ν(O-H): 3426 cm^−1^; ν(CH=N): 1583cm^−1^; ν(C=C): 1427, 1458, 1474, 1518 cm^−1^; ν(C–O): 1296 cm^−1^; ν(O-Cu): 667cm^−1^; ν(N-Cu): 626 cm^−1^. ^1^H NMR (DMSO-d_6_, 500 MHz, δ in ppm): 0.93 (s, 6H, (CH_3_)_2_), 3.43 (s, 4H, (CH_2_)_2_), 6.84 (t, 4H, Ar-H, J_o_ = 7.23 Hz), 7.28 (td, 2H, Ar-H, J_o_ = 8.51 Hz, J_m_ = 1.69 Hz), 7.40 (dd, 2H, Ar-H, J_o_ = 7.60 Hz, J_m_ = 1.73 Hz), 8.50 (s, 1H, CH=N), 13.60 (s, 1H, Ar–OH). ^13^C NMR (DMSO-d_6_, 125 MHz, δ in ppm): 8.96 ((CH_3_)_2_), 36.29 (C(CH_3_)_2_), 45.82 ((CH_2_)_2_), 117.00, 119.09 (C-CH=N), 132.23, 132.92, 161.25 (C-OH), 167.15 (CH=N). UV–Vis (DMSO, λ_max_ in nm, (ε_max_ in mol·L^−1^·cm^−1^): 267 (7261), 317 (5492); 408 (1524); 602 (610). HRMS [M+H]^+^: calcd: 482.01018, obtained: 483.75846. Anal. Calcd for C_16_H_14_N_2_O_2_Cl_2_Ru: C, 47.31; H, 4.60; N, 5.81; O, 6.63. Found: C, 47.21; H, 4.29; N, 5.45; O, 6.34.

### 3.3. In Vitro Antioxidant Activity Assays

The demonstration of the antioxidant power of the obtained compounds has been evaluated by two methods: the free radical 2,2-diphenyl-1-picrylhydrazyl (DPPH) scavenging test and the ferric reducing-antioxidant power (FRAP) assay.

#### 3.3.1. DPPH Radical Scavenging Method

The DPPH free radical scavenging is based on the ability of a compound to reduce the radical DPPH^•^. The reduction results are identified by the change in the color of the solution that turns from purple to yellow in the presence of an antiradical compound. The ability of the products to reduce the DPPH radical was determined according to the method reported by Blois [28]. Thus, 1 mL of the tested compounds in DMSO at different concentrations (0.05, 0.1, 0.3, 0.5, 0.7, and 1 μg/mL) was added to 2.5 mL of the methanolic solution of DPPH (24 mg/L). After 30 min of incubation in darkness at room temperature, the absorbance was read at 517 nm against a blank using a UV-VIS spectrophotometer, and the concentration necessary to degrade 50% of the DPPH radical (IC_50_) was determined. Ascorbic acid was used as a standard.

#### 3.3.2. FRAP Method

The reducing power of the obtained compounds is determined by the FRAP method [29]. This method is grounded on the reduction of the ferric ion (Fe^3+^) into ferrous ions (Fe^2+^). Therefore, 1 mL of the sample at different concentrations was mixed with 2.5 mL of phosphate buffer (0.2 M, pH 6.6) and 2.5 mL of potassium hexacyanoferrate [K_3_Fe(CN)_6_] at 1%. After incubation of the mixture at 50 °C for 20 min, 2.5 mL of 10% trichloroacetic acid was added to stop the reaction, and after the centrifugation of the tubes, 2.5 mL of the supernatant of each concentration was mixed with 2.5 mL of distilled water and 0.5 mL of a 0.1% FeCl_3_ solution and left at rest protected from light for 30 min before measuring the absorbance at 700 nm against a blank. Ascorbic acid is used as a positive control, the absorbance of which was measured under the same conditions as the samples tested. The increase in absorbance corresponds to an increase in the reducing power of the tested compounds.

#### 3.3.3. Total Antioxidant Capacity (TAC)

The PPM (PhosphoMolybdate) test is a variant of the DPPH test. In this test, hydrogen and electrons are transferred from the reducing compound (tested compound) to the oxidizing complex (PPM). This transfer depends on the redox potential, the pH of the medium, and the structure of the antioxidant compound.

The total antioxidant capacity (TAC) of the ligand and its metal complexes are evaluated by the Phosphomolybdenum method. This technique is based on the reduction of molybdenum Mo(VI) present in the form of molybdate ions MoO_4_^2-^ to molybdenum Mo(V) MoO^2+^ in the presence of the tested compounds. The green phosphate/Mo(V) complex forms at acidic pH [30].

A volume of 0.3 mL of each concentration was mixed with 3 mL of reagent solution (0.6 M sulfuric acid, 28 mM sodium phosphate, and 4 mM molybdate ammonium). The tubes were screwed and incubated at 95 °C for 90 min. After cooling, the absorbance of the solutions was measured at 695 nm against the blank which contained 3 mL of the solution of the reagent and 0.3 mL of DMSO and was incubated under the same conditions as the sample.

### 3.4. In Vitro Antibacterial Activity

#### 3.4.1. Pathogen Bacteria and Growth Conditions

Antibacterial activity screening of [RuL^1^Cl_2_], [RuH_2_L^2^Cl_2_], and [RuH_2_L^3^Cl_2_] was performed against pathogenic bacteria, obtained from the Laboratory of Biology and Health, Faculty of Sciences of Tetouan, including Gram-positive bacteria (*Bacillus subtilis* DSM 6633, and *Staphylococcus aureus* CECT 976) and Gram-negative bacteria [*Proteus mirabilis* INH, *Escherichia coli* K12, and *Pseudomonas aeruginosa* CECT 118), using the disc diffusion method as described in our previous research work [31]. First, sterile discs (6 mm diameter) applied onto the surface of the MHA, which was previously spread with the test inoculum concentrations, were loaded with a volume of 20 µL of each product at 50 mg/mL. Gentamicin (15 µg) served as a positive control, and 10% of dimethylsulfoxide (DMSO) served as a negative control. After the incubation, the antibacterial effect was determined by calculating the diameter of inhibition zones.

#### 3.4.2. Minimum Inhibitory Concentration and Minimum Bactericidal Concentration

The MIC values of active products were evaluated in a sterile 96-well microplate according to Benali et al. [32] with some modifications. First, except for the first well with a volume of 200 μL containing each tested product with a concentration of 25 mg/mL in 10% DMSO, 100 μL of Mueller–Hinton Broth (MHB) was distributed in all test wells. A series of doses varying from 0.097 to 25 mg/mL were prepared from the first to the ninth well. Then, 10 μL of the suspension from each well was removed and replaced by the inoculum test concentration, except the 10th well used as a sterility control. The eleventh and twelfth wells were considered as positive growth and negative controls containing only MHB broth and 10% DMSO (*v*/*v*) without tested compounds, respectively. Then, the plates were incubated at 37 °C for 24h. After the incubation, a volume of 25 μL of an indicator of microorganism growth was added to each well; 2,3,5-triphenyltetrazolium chloride (TTC) was prepared at a concentration of 5 mg/mL in sterile distilled water. The microplate was re-incubated for 30 min at a temperature of 37 °C. The minimum bactericidal concentration (MBC) was determined by the inoculation in MHA of 10 μL of broth from the uncolored wells and incubated for 24 h at 37 °C.

## 4. Conclusions

In summary, in this work, we were able to the synthesize novel Ru(II) complex-based salen Schiff bases. The structures of the synthesized Ru(II) complexes were well established using spectroscopic, spectrometric, and thermal analysis techniques. The data show that the new Ru(II) complexes exhibit an octahedral geometry. The DPPH free radical scavenging, FRAP, and total antioxidant capacity (TAC) assays show that the screened complexes display a promising antioxidant activity and they are more potent than the used positive control. The Ru(II) complexes also exhibit promising antibacterial responses against pathogen bacteria including Gram-positive and Gram-negative strains.

## Figures and Tables

**Table 1:** Crystallographic data for Ru(II) complexes.

Complexes	[RuL^1^Cl_2_]	[RuH_2_L^2^Cl_2_]	[RuH_2_L^3^Cl_2_]
Formula	C_16_H_14_Cl_2_RuN_2_O_2_	C_17_H_18_Cl_2_RuN_2_O_2_	C_19_H_22_Cl_2_RuN_2_O_2_
MW	437.95	453.97	482.01
System	Monoclinic	Monoclinic	Monoclinic
Space Group	C 1 2/c 1	P 1 21/c 1	P 1 21 1
T(K)	296	296	296
λ (Å)	1.54060	1.54060	1.54060
a/	13.4890	16.0430	7.1520
b/	9.6910	12.9140	10.7120
c/	16.6850	16.5030	9.9590
α/^o^	90.0000	90.0000	90.0000
β/^o^	112.3650	114.7750	91.2000
γ/^o^	90.0000	90.0000	90.0000
V (Å^3^)	762.81	3104.39	762.81
Density (g/cm^3^)	1.71	1.35	1.89
Radiation	CuKα	CuKα	CuKα
Scan range 2θ (°)	10–80	10–80	10–80

**Table 2:** IC_50_ values for DPPH radical scavenging activity of tested compounds.

Compounds	H_2_L^1^	H_2_L^2^	H_2_L^3^	[RuL^1^Cl_2_]	[RuH_2_L^2^Cl_2_]	[RuH_2_L^3^Cl_2_]	Ascorbic Acid
IC50 ± SD (μg/mL)	-	0.99 ± 0.01	-	0.09 ± 0.03	0.15 ± 0.03	0.04 ± 0.01	0.14 ± 0.01

**Table 3:** Antibacterial activity of products determined by the disc diffusion method, and the minimum inhibitory concentration (MIC) and minimum bactericidal concentration (MBC) (mg/mL) of products.

	[RuL^1^Cl_2_]			[RuH_2_L^2^Cl_2_]			[RuH_2_L^3^Cl_2_]		
	DIZ *	MIC^+^	MBC^−^	DIZ *	MIC	MBC	DIZ *	MIC	MBC
*S. aureus*	na	nt	nt	na	nt	nt	na	nt	nt
*B. subtilis*	na	nt	nt	21 ± 1.41	0.97	0.97	17 ± 1.41	0.39	0.39
*P. aeruginosa*	na	nt	nt	na	nt	nt	na	nt	nt
*P. mirabilis*	na	nt	nt	25.5 ± 0.70	0.97	0.97	16.50 ± 0.70	0.78	0.78
*E. coli*	na	nt	nt	na	nt	nt	na	nt	nt

* The diameters of the inhibition zones (mm) are given as mean ± SD of duplicate experiments; na: no activity; MIC^+^: minimum inhibitory concentration; MBC^−^: minimum bactericidal concentration; nt: not tested.

## Data Availability

All additional data analyzed during this study can be found in the “Supplementary Materials” section of this article.

## Supplementary Materials

The following supporting information can be downloaded at https://www.mdpi.com/article/10.3390/ijms25147879/s1.
