# Supplementary material for: Ruthenium(II) Complex-Based Tetradentate Schiff Bases: Synthesis, Spectroscopic, Antioxidant, and Antibacterial Investigations
Source: Int J Mol Sci. 2024 Jul 18;25(14):7879. doi: 10.3390/ijms25147879 (PMC11277530; doi:10.3390/ijms25147879)
Supplement: Supplementary file 1 [file ijms-25-07879-s001.zip › ijms-3063650-supplementary.pdf]

Supplementary Materials

Ruthenium(II) Complex-Based Tetradentate Schiff Bases: Synthesis, Spectroscopic, Antioxidant, and Antibacterial Investigations

Bouchra Es-Sounni [1], Kaoutar Harboul [2], Ayoub Mouhib [3], Ashwag S. Alanazi [4], Mohamed Hefnawy [5], Mohamed Bakhouch [3,*], Taoufiq Benali [2,6], Khalil Hammani [2], Noureddine Mazoir [3], Mohamed El Yazidi [7], Ahmed Benharref [8] and Mohammed Fahim [1]

1 Laboratory of Innovative Materials and Biotechnology of Naturel Resources, Faculty of Sciences, Moulay Ismail University, Meknes 50000, Morocco; bouchrasounni@gmail.com (B.E.-S.)
2 Laboratory of Natural Resources and Environment, Polydisciplinary Faculty of Taza, Sidi Mohamed Ben Abdellah University of Fez, Taza 30050, Morocco; benali.taoufiq@gmail.com (T.B.); khalil.hammani@usmba.ac.ma (K.H.)
3 Bioorganic Chemistry Team, Department of Chemistry, Faculty of Sciences, Chouaïb Doukkali University, El Jadida 24000, Morocco
4 Department of Pharmaceutical Sciences, College of Pharmacy, Princess Nourah Bint Abdulrahman University, Riyadh 1167, Saudi Arabia; asalanzi@pnu.edu.sa
5 Department of Pharmaceutical Chemistry, College of Pharmacy, King Saud University, Riyadh 11451, Saudi Arabia; mhefnawy@ksu.edu.sa
6 Environment and Health Team, Polydisciplinary Faculty of Safi, Cadi Ayyad University, Safi 46000, Morocco
7 Engineering Laboratory of Organometallic and Molecular Materials and Environment, Faculty of Sciences Dhar El Mahraz, University Sidi Mohamed Ben Abdellah, Fez 30000, Morocco; elyazidimohamed@hotmail.com
8 Laboratory of Natural Substances Chemistry, Faculty of Sciences Semlalia, Cadi Ayyad University, Marrakech 40000, Morocco; a.benharref@gmail.com
* Correspondence: bakhouch.m@ucd.ac.ma

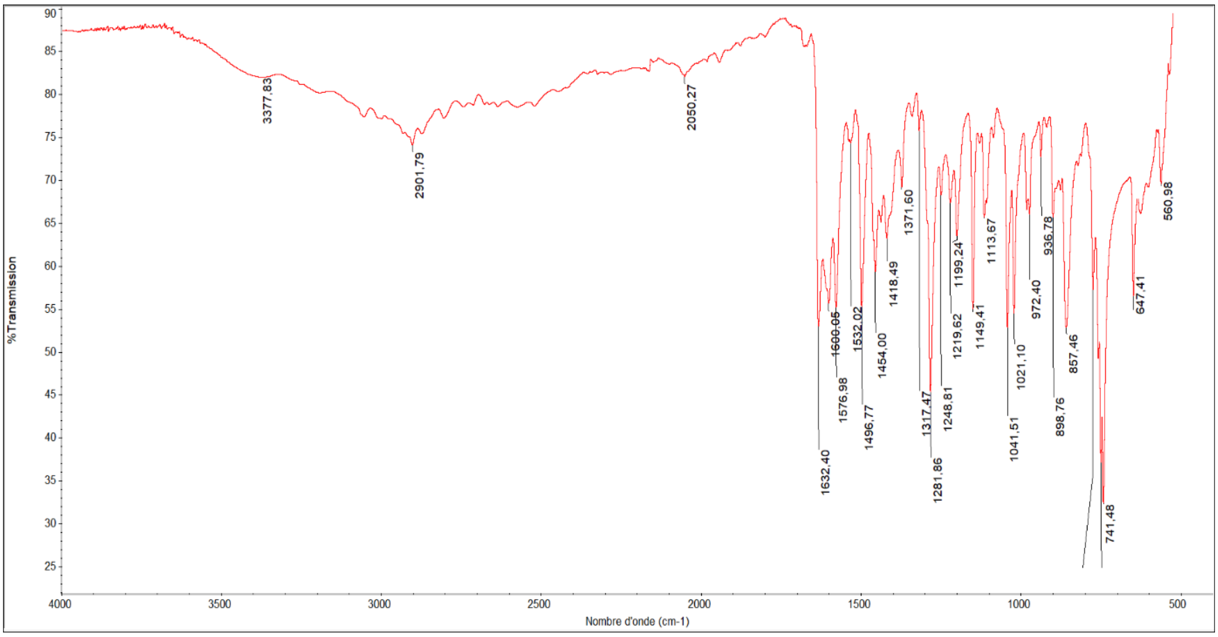

Figure S1. ATR-IR spectrum of H_2L^1.

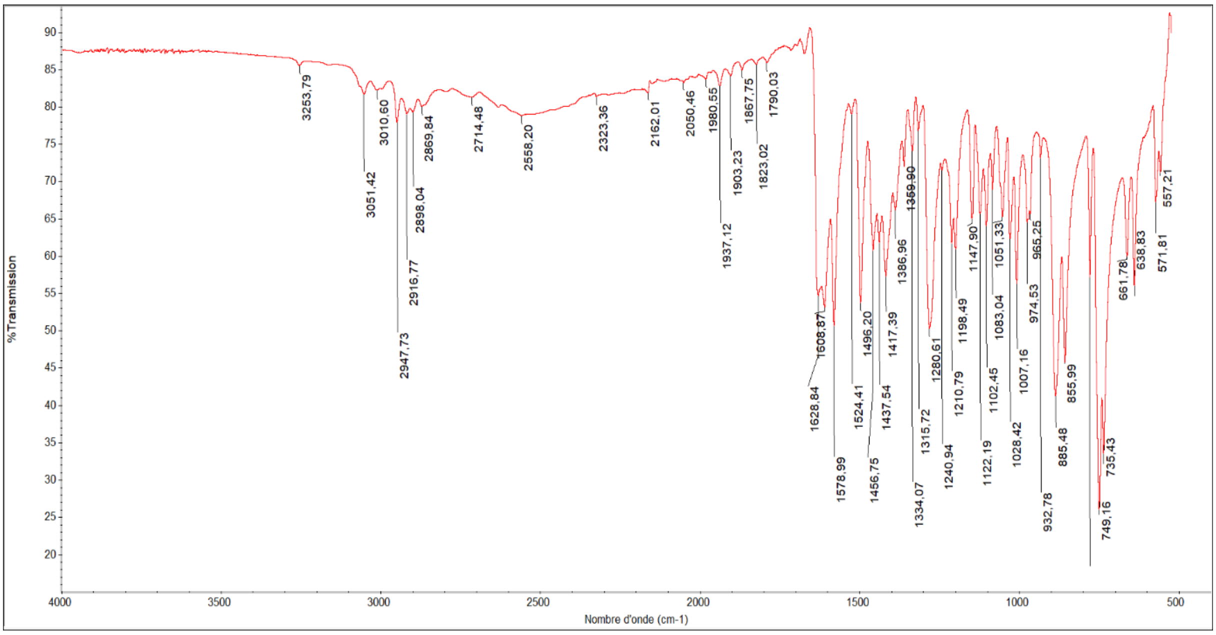

Figure S2. ATR-IR spectrum of H_2L^2.

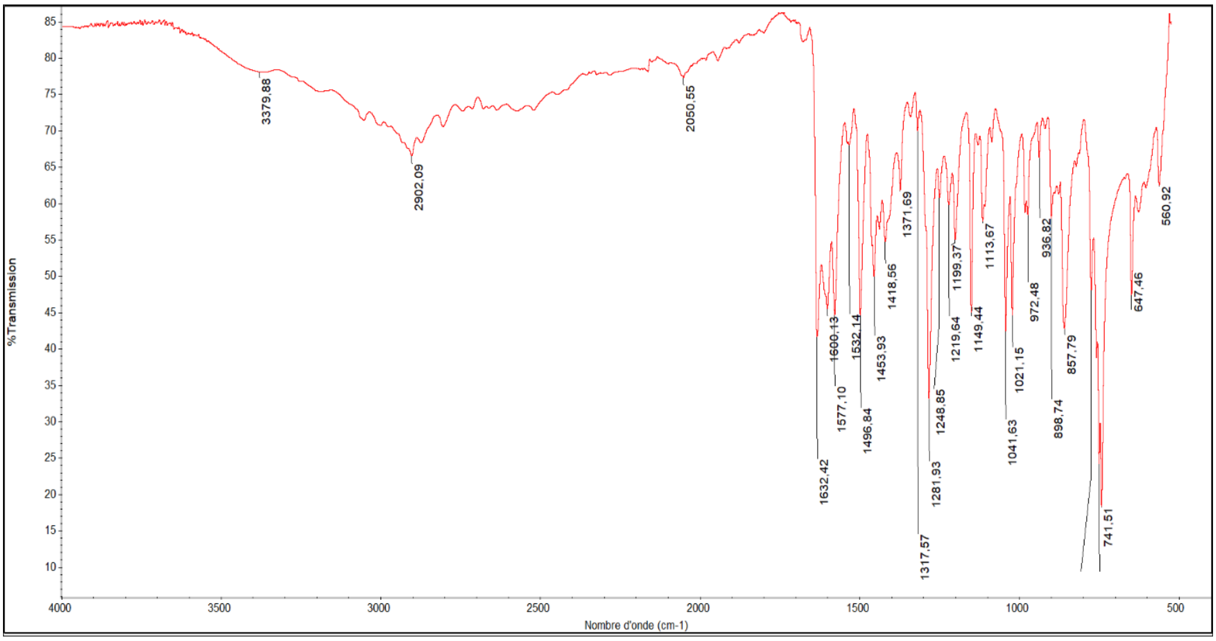

Figure S3. ATR-IR spectrum of H_2L^3.

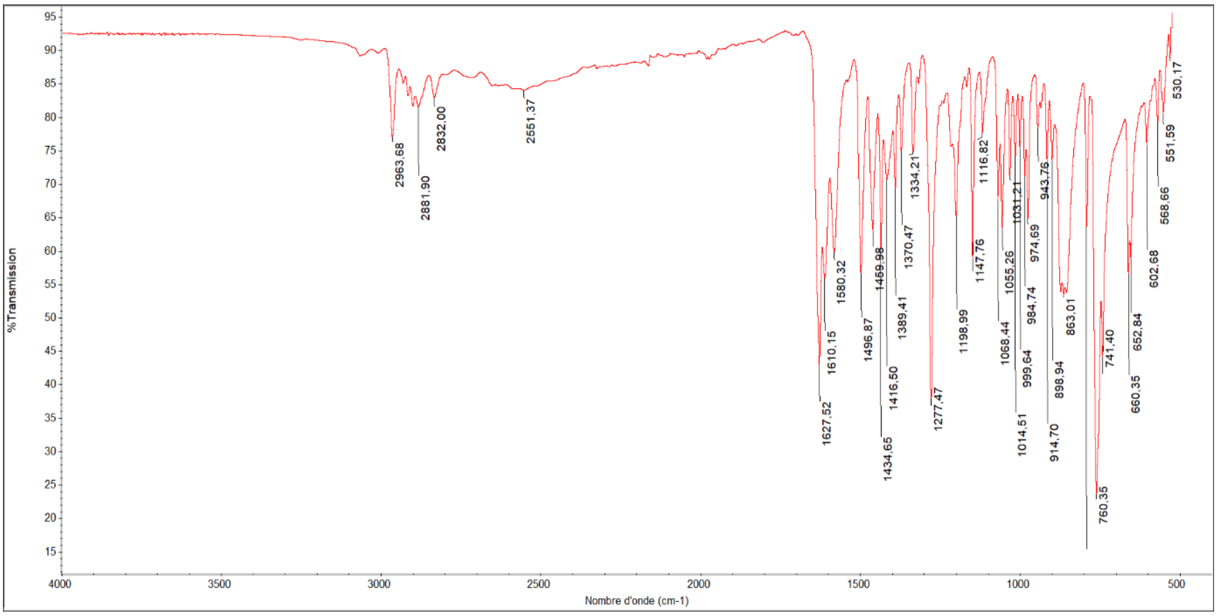

Figure S4. ATR-IR spectrum of $[RuL^1Cl_2]$.

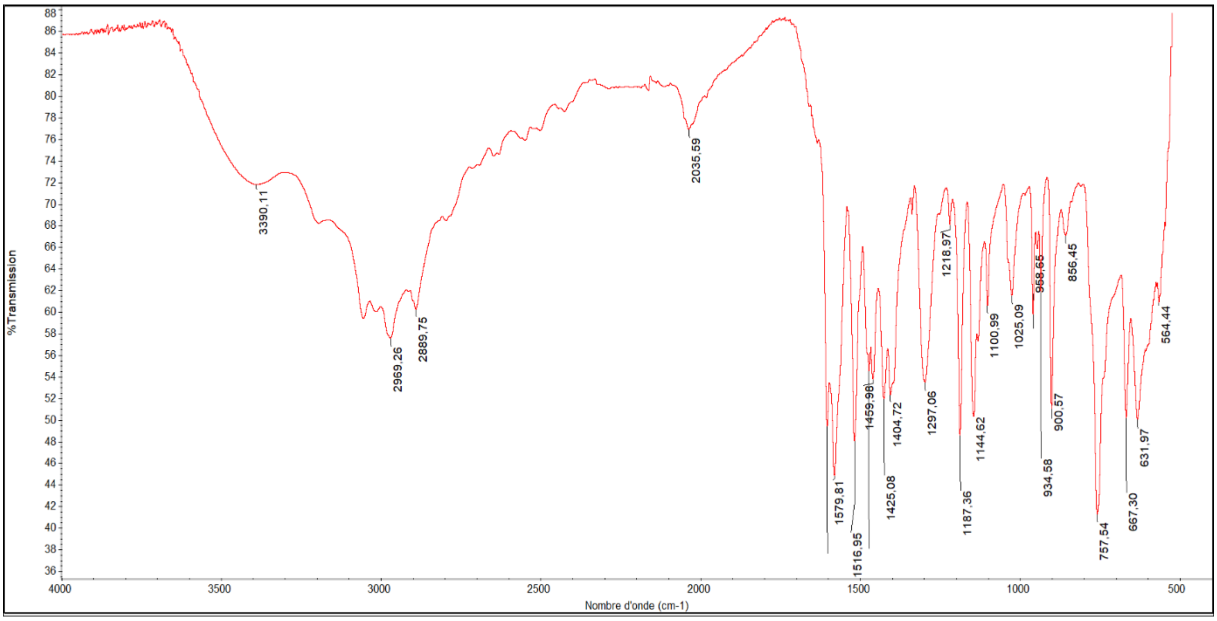

Figure S5. ATR-IR spectrum of [$RuH_2L^2Cl_2$].

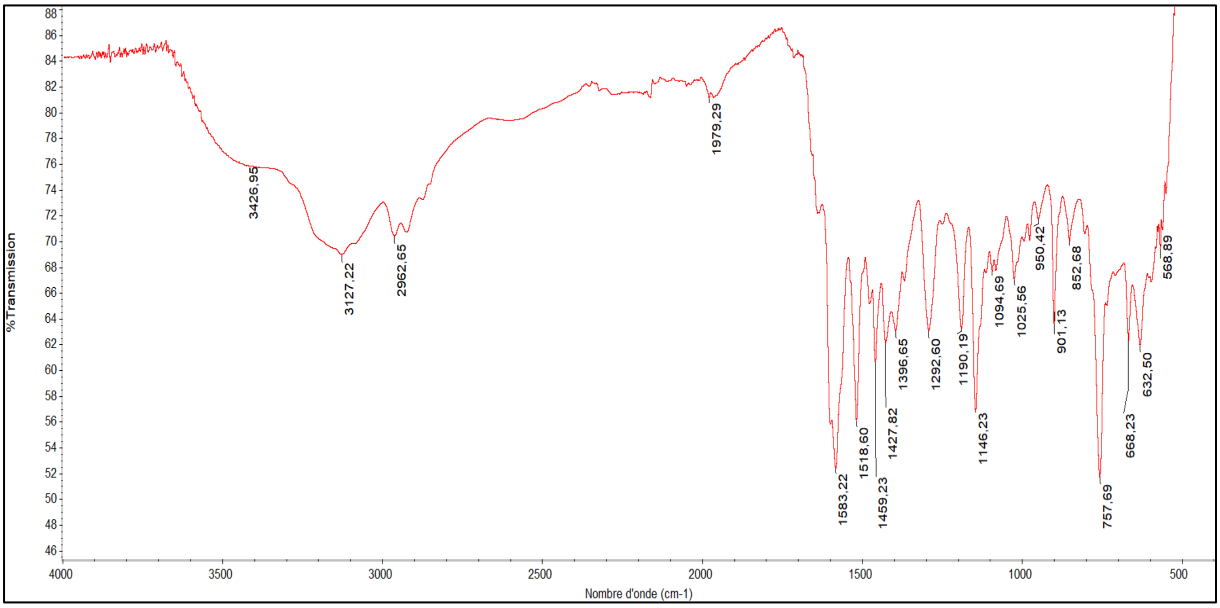

Figure S6. ATR-IR spectrum of [$RuH_2L^3Cl_2$].

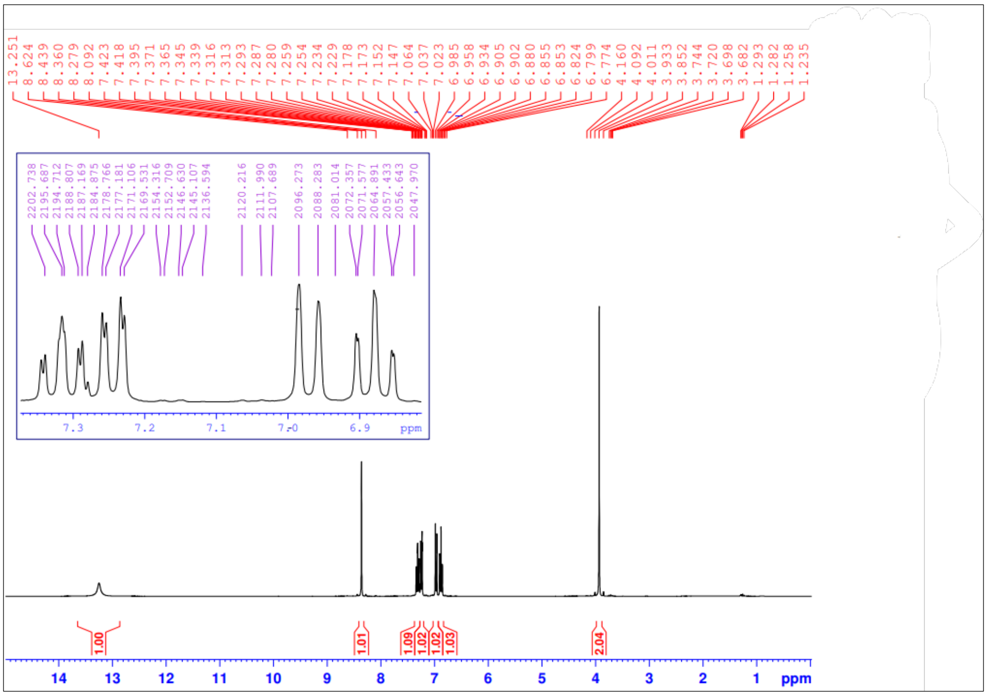

Figure S7. ^{1}H NMR (300 MHz) spectrum of H_2L^1.

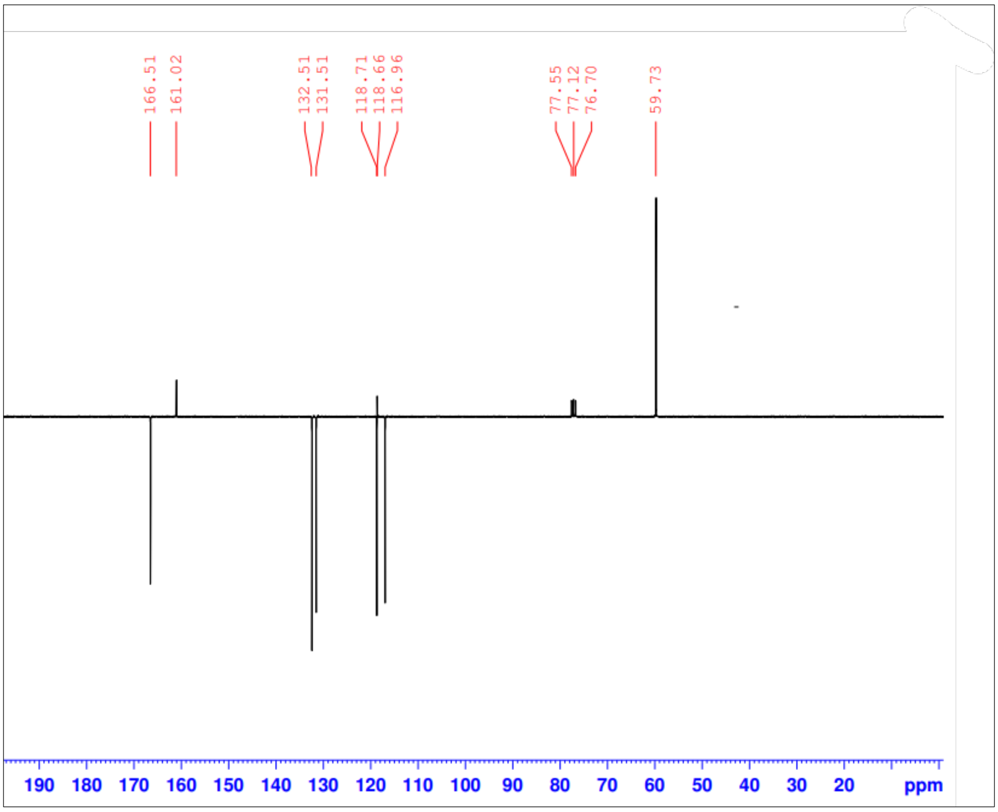

Figure S8. ^{13}C NMR (APT, 75 MHz) spectrum of H_2L^1.

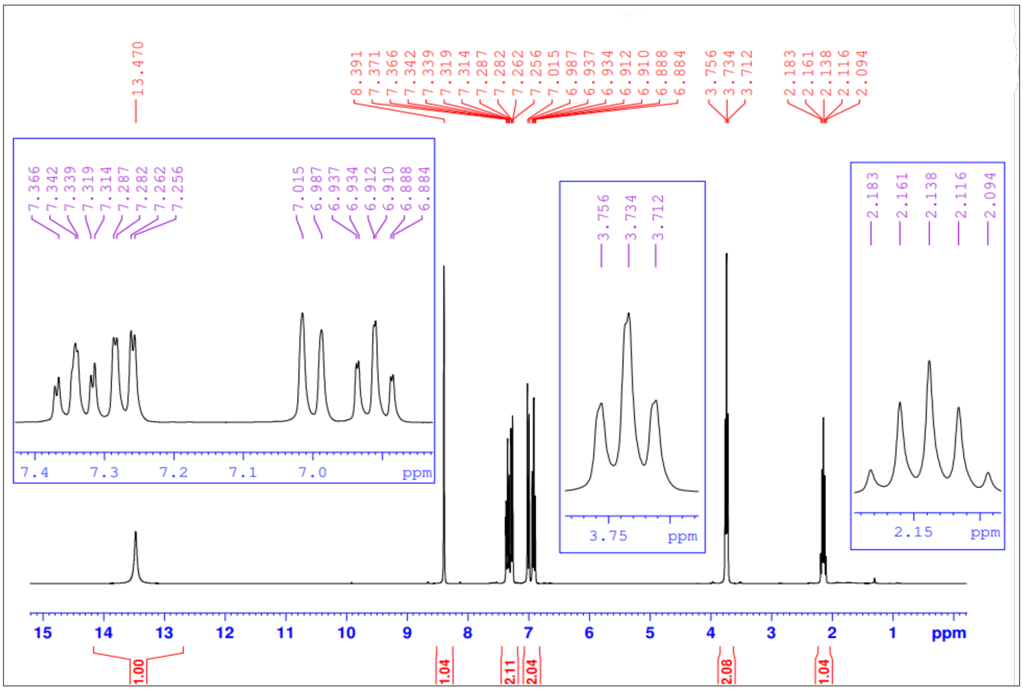

Figure S9. ^{1}H NMR (300 MHz) spectrum of H_2L^2.

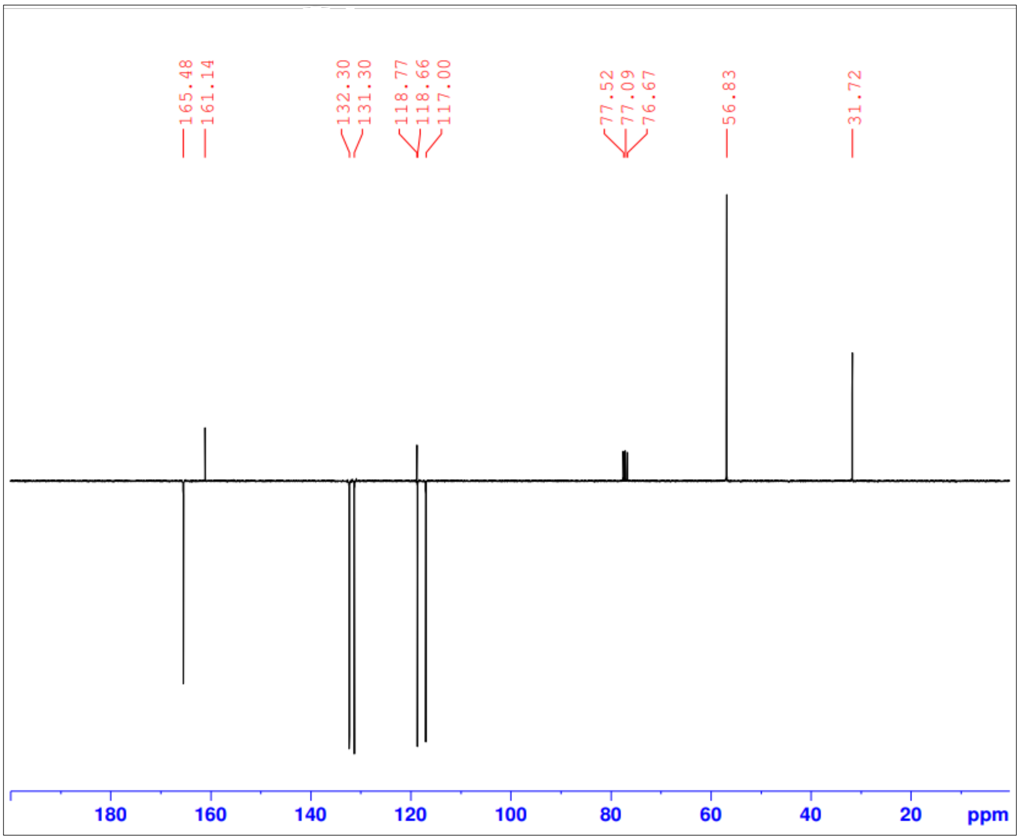

Figure S10. ^{13}C NMR (APT, 75 MHz) spectrum of H_2L^2.

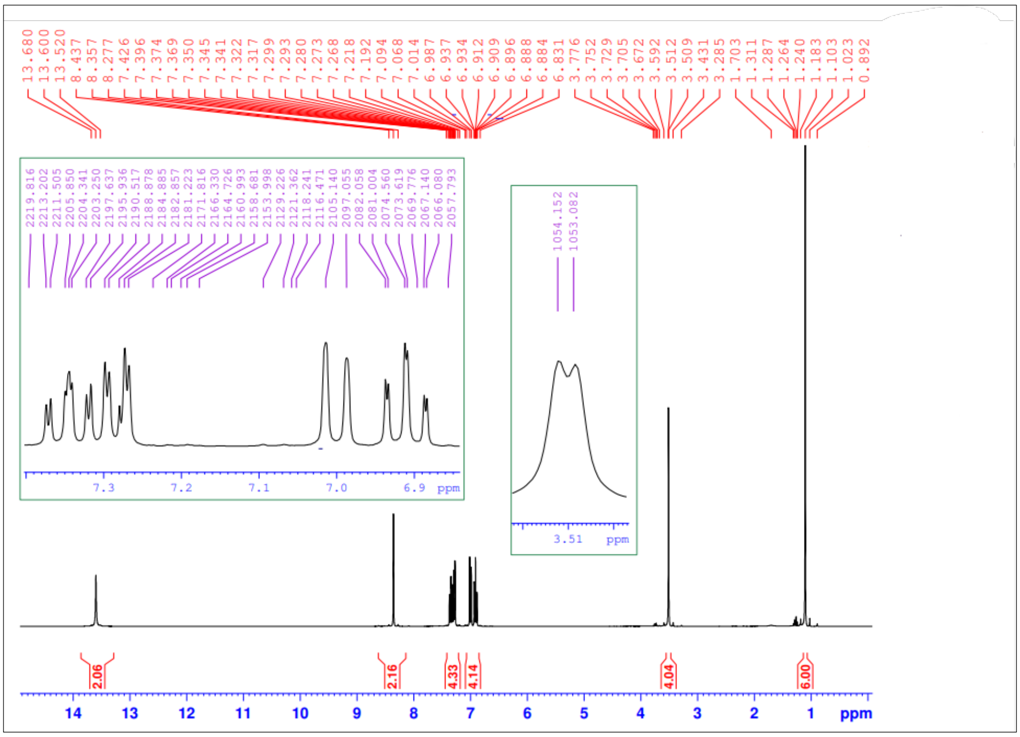

Figure S11. ^{1}H NMR (300 MHz) spectrum of H_2L^3.

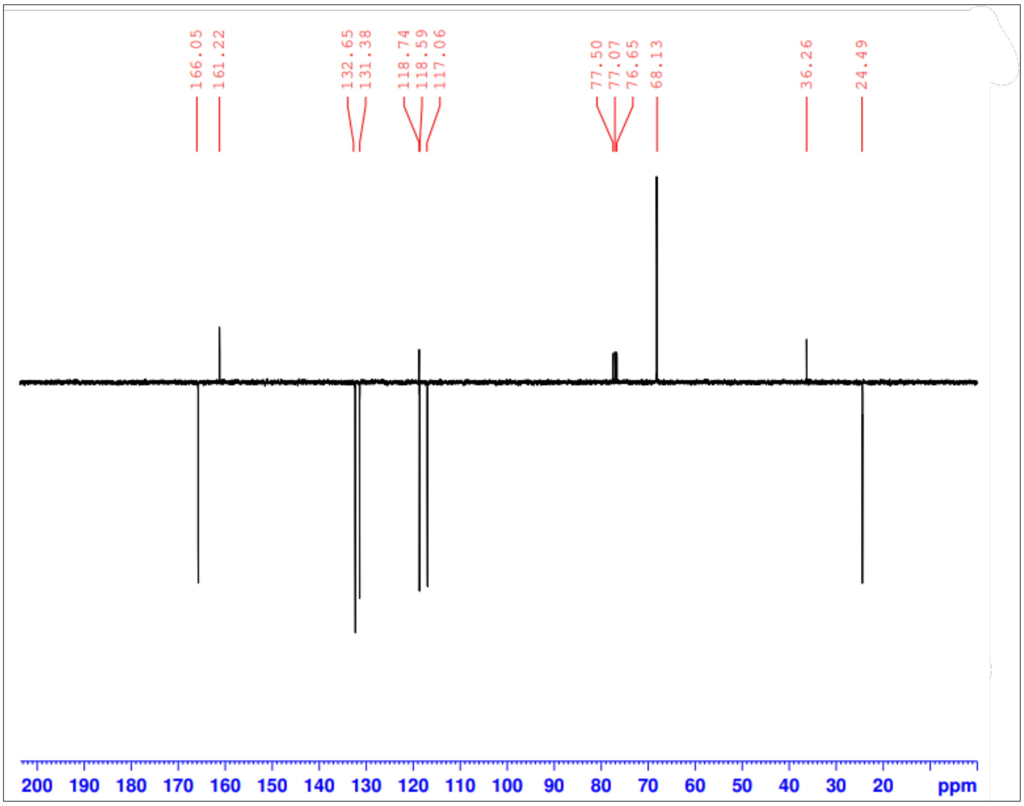

Figure S12. ^{13}C NMR (APT, 75 MHz) spectrum of H_2L^3.

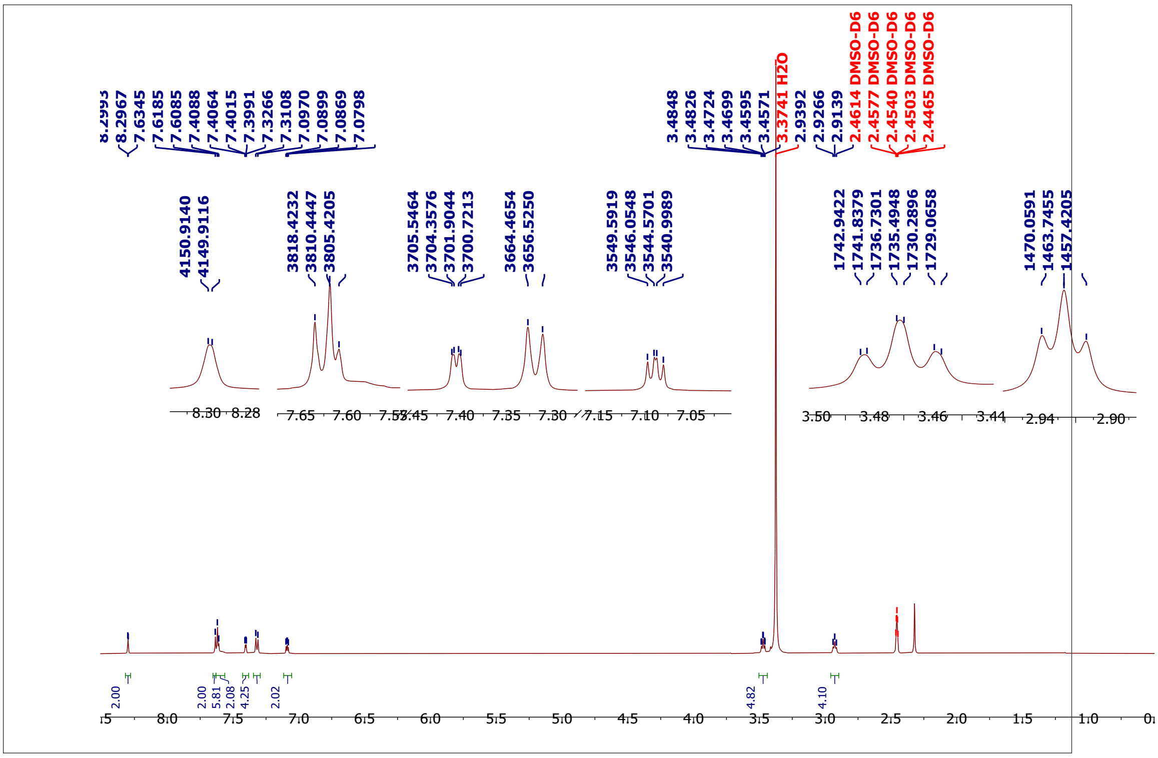

Figure S13. ^{1}H NMR (500 MHz) spectrum of [$RuL^{1}Cl_2$].

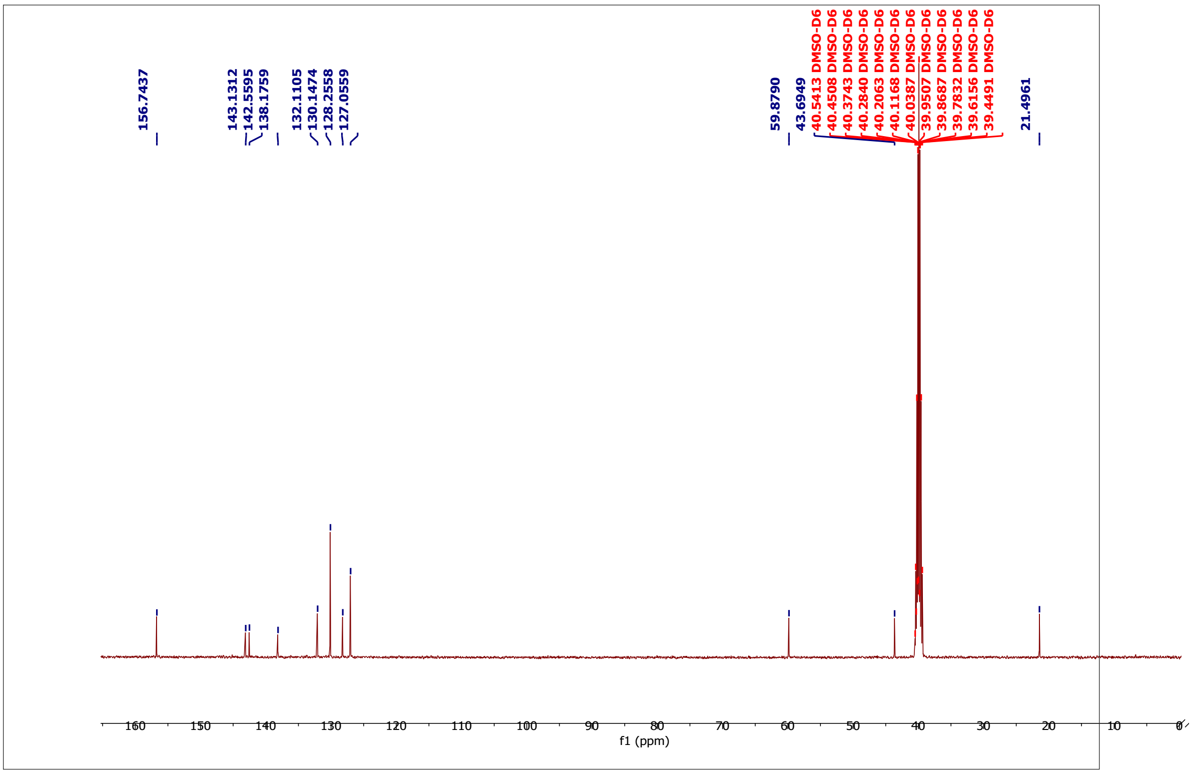

Figure S14. ^{13}C NMR (125 MHz) spectrum of [$RuL^{1}Cl_2$].

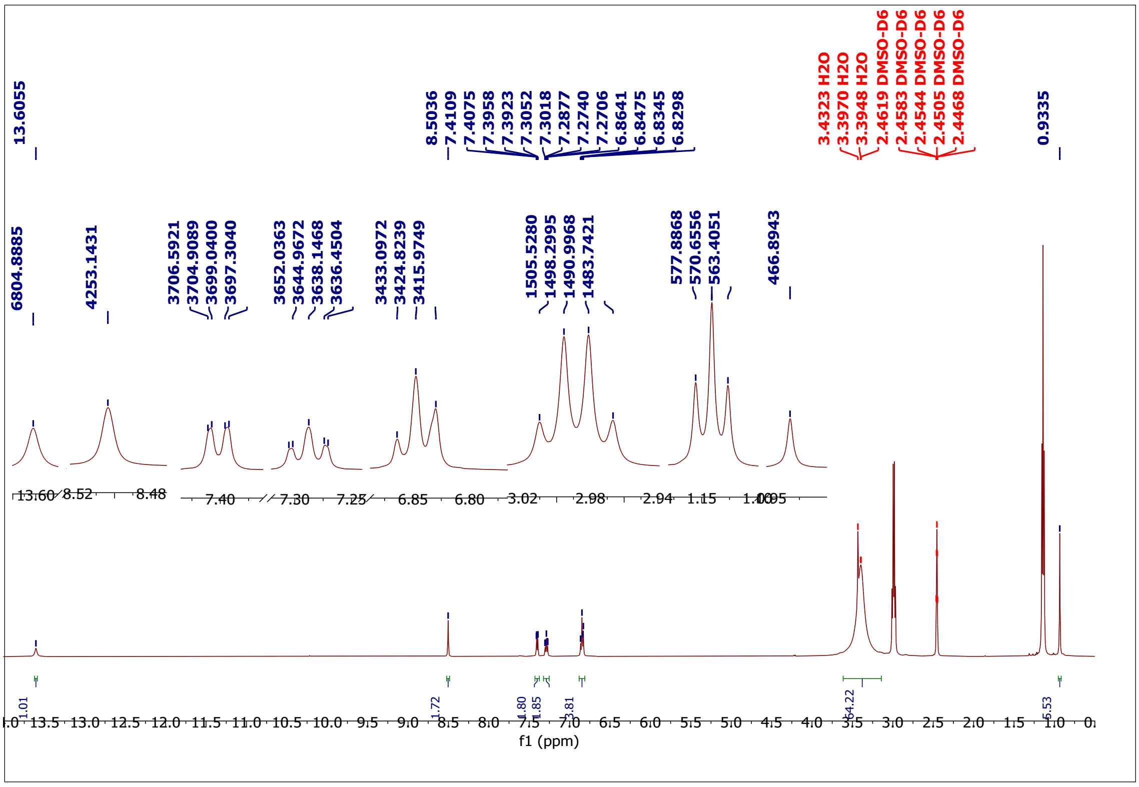

Figure S15. ^{1}H NMR (500 MHz) spectrum of $[RuH_2L^3Cl_2]$.

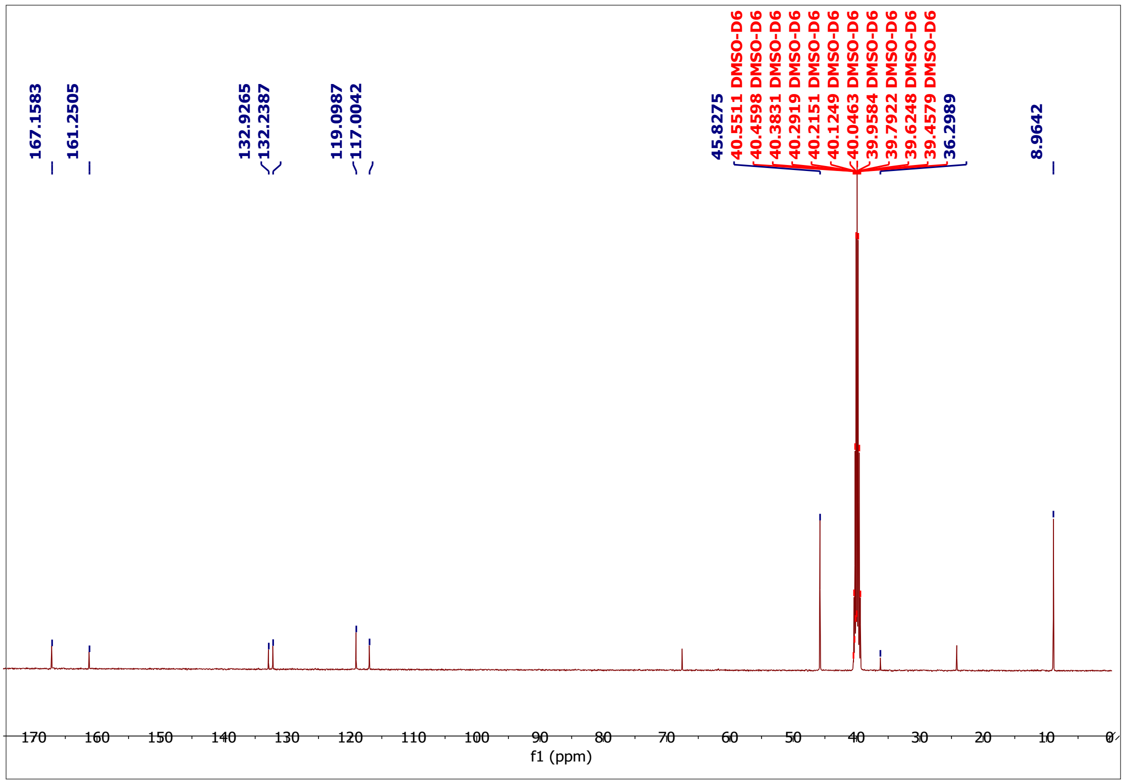

Figure S16. ^{13}C NMR (125 MHz) spectrum of $[RuH_2L^3Cl_2]$.

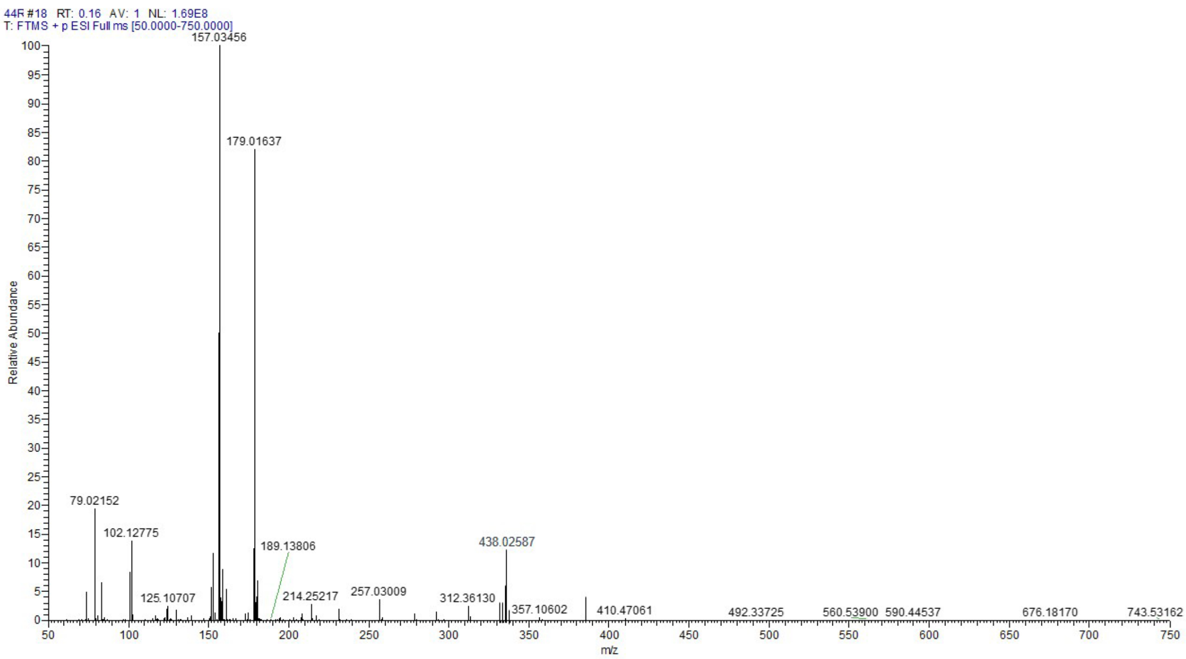

Figure S17. HRMS spectrum of [RuL^{1}Cl$_2$].

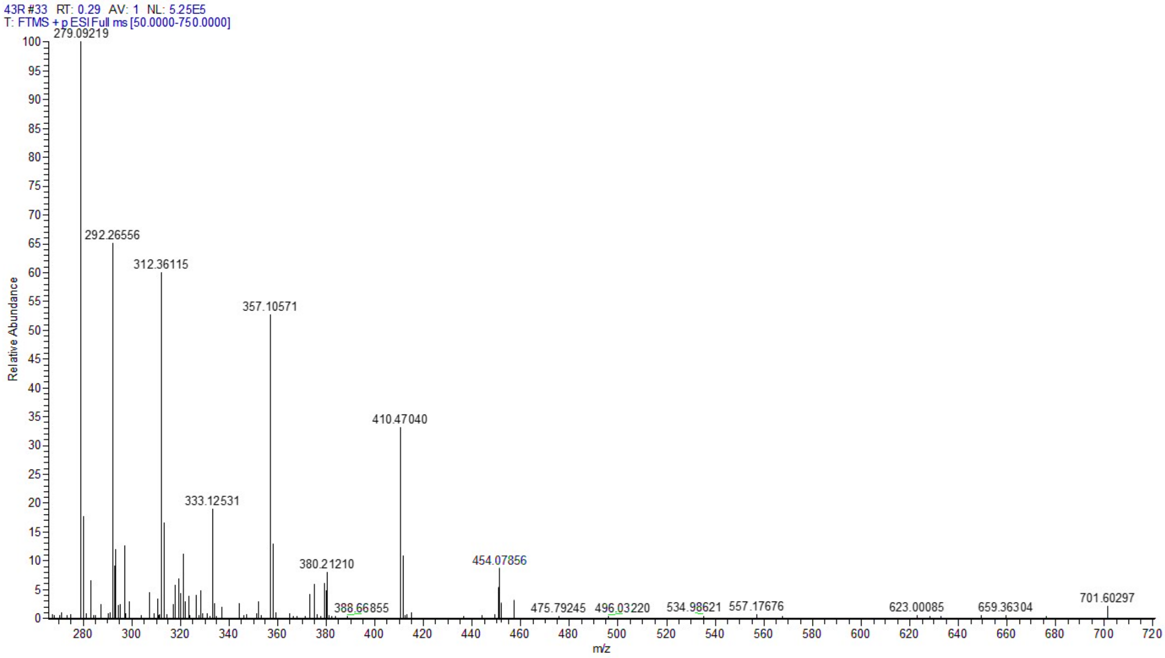

Figure S18. HRMS spectrum of [RuH$_2$L^{2}Cl$_2$].

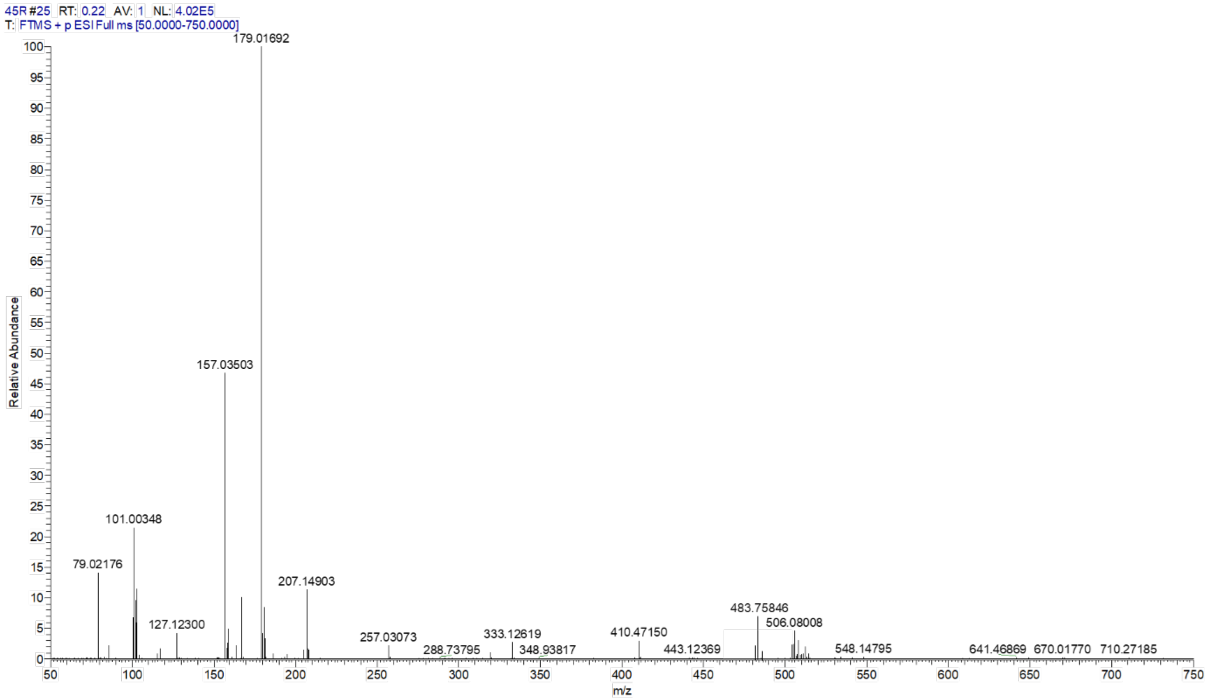

Figure S19. HRMS spectrum of [RuH$_2$L^{3}Cl$_2$].
